# In vitro-generated human muscle reserve cells are heterogeneous for Pax7 with distinct molecular states and metabolic profiles

**DOI:** 10.1186/s13287-023-03483-5

**Published:** 2023-09-08

**Authors:** Axelle Bouche, Benoit Borner, Chloé Richard, Ysaline Grand, Didier Hannouche, Thomas Laumonier

**Affiliations:** 1 Cell Therapy and Musculoskeletal Disorders Laboratory, Department of Orthopedic Surgery, Geneva University Hospitals and Faculty of Medicine, University Medical Center, 1 rue Michel Servet, 1211 Geneva, Switzerland; 2 Department of Cell Physiology and Metabolism, Faculty of Medicine, Geneva, Switzerland

**Keywords:** Human muscle reserve cell, Pax7 heterogeneity, Quiescence, AMPK, Metabolism, Fatty acid oxidation

## Abstract

**Background:**

The capacity of skeletal muscles to regenerate relies on Pax7^+^ muscle stem cells (MuSC). While in vitro-amplified MuSC are activated and lose part of their regenerative capacity, in vitro-generated human muscle reserve cells (MuRC) are very similar to quiescent MuSC with properties required for their use in cell-based therapies.

**Methods:**

In the present study, we investigated the heterogeneity of human MuRC and characterized their molecular signature and metabolic profile.

**Results:**

We observed that Notch signaling is active and essential for the generation of quiescent human Pax7^+^ MuRC in vitro. We also revealed, by immunofluorescence and flow cytometry, two distinct subpopulations of MuRC distinguished by their relative Pax7 expression. After 48 h in differentiation medium (DM), the Pax7^High^ subpopulation represented 35% of the total MuRC pool and this percentage increased to 61% after 96 h in DM. Transcriptomic analysis revealed that Pax7^High^ MuRC were less primed for myogenic differentiation as compared to Pax7^Low^ MuRC and displayed a metabolic shift from glycolysis toward fatty acid oxidation. The bioenergetic profile of human MuRC displayed a 1.5-fold decrease in glycolysis, basal respiration and ATP-linked respiration as compared to myoblasts. We also observed that AMPKα1 expression was significantly upregulated in human MuRC that correlated with an increased phosphorylation of acetyl-CoA carboxylase (ACC). Finally, we showed that fatty acid uptake was increased in MuRC as compared to myoblasts, whereas no changes were observed for glucose uptake.

**Conclusions:**

Overall, these data reveal that the quiescent MuRC pool is heterogeneous for Pax7 with a Pax7^High^ subpopulation being in a deeper quiescent state, less committed to differentiation and displaying a reduced metabolic activity. Altogether, our data suggest that human Pax7^High^ MuRC may constitute an appropriate stem cell source for potential therapeutic applications in skeletal muscle diseases.

**Supplementary Information:**

The online version contains supplementary material available at 10.1186/s13287-023-03483-5.

## Background

Skeletal muscle is one of the most plastic tissues of the human body due to its ability to repair after tissue damage. This strong regenerative capability depends mainly on adult muscle stem cells (MuSCs) also known as satellite cells [1–3]. They represent, depending on species, age, muscle location and muscle type, around 5% of skeletal muscle nuclei [4] and exist in a reversible non-dividing state of quiescence under homeostatic conditions. In response to injury, MuSCs activate, enter the cell cycle, proliferate and differentiate to repair damaged muscle fibers, while a small proportion of MuSCs return to quiescence for future needs of regeneration [5]. Quiescent MuSCs are characterized by the expression of the transcription factor Pax7, known to play a major role in regulating the myogenic identity and function of MuSCs [2, 6]. The quiescent state of MuSCs is both actively maintained and dynamically regulated [7], and Notch signaling is known to be essential in the regulation and maintenance of MuSCs quiescence [8–11]. Through RNA sequencing, it was described that quiescent murine MuSCs are molecularly and phenotypically heterogeneous [12, 13]. Using Pax7 transgenic mice, Rocheteau et al*.* [14] demonstrated that a subpopulation of MuSCs, expressing higher levels of Pax7 (Pax7^High^), represents a dormant stem cell population with improved regenerative capacities. Heterogeneity was also documented within the human MuSC pool with identification of human Pax7 + MuSCs subpopulations with distinct functionalities [15, 16]. MuSC heterogeneity was also described thanks to other specific markers. Recently, Ma et al. demonstrated that MuSCs are also morphologically heterogeneous with different patterns of cellular protrusions that allow to classify MuSC in three subtypes: responsive, intermediate and sensory cells [17]. MuSCs are also heterogeneous for the expression of a primary cilium [18], a structure that protrudes from quiescent MuSCs and which resorbs rapidly upon activation [19]. It was recently demonstrated that primary cilium play an important role to maintain MuSCs in a G0 dormant state [20] and can regulate the ability of MuSCs to regenerate [21].

MuSCs display also a flexible metabolism that adapts to quiescence, activation and differentiation states [22, 23]. Quiescent MuSCs are mostly dependent on oxidative phosphorylation (OXPHOS) and fatty acid oxidation (FAO), while, upon activation, quiescent MuSCs operate a metabolic shift from FAO toward glycolysis [5, 24, 25]. FAO appears necessary to maintain MuSCs in a quiescent state and its modulation can have major consequences on cell function and may be targeted to control MuSCs fate in therapeutic strategies [5].

MuSCs have long been explored as a potential therapeutic approach for muscular dystrophies as they would allow genetic complementation and restore the regenerative capacity of damaged muscles. However, despite considerable technical and scientific progress, the outcome of this therapeutic approach has not met the initial hopes [26–28]. It is now well-established that freshly isolated MuSCs grown in conventional 2D culture conditions ex vivo are quickly pulled out of quiescence [24], which correlates with a drastic diminution of their regenerative potential [29]. This issue is not restricted to MuSCs, as it concerns most stem cell types dissociated and amplified in vitro*.* Recent efforts have focused on optimizing *ex vivo* conditions that permit the expansion of MuSCs while maintaining them in an undifferentiated state and preserving their high regenerative potency following in vivo engraftment [8–10, 30]. Nevertheless, these protocols, developed in animal models, are difficult to apply to clinical situations. Recently, we demonstrated that human muscle reserve cells (MuRC), generated in vitro, are quiescent Pax7^+^ myogenic stem cells very similar to MuSCs with properties compatible for their use in cell therapy [31, 32]. However, the heterogeneity of human MuRC subpopulations is not well-described, especially their molecular signature and metabolic profile. For this purpose, we evaluated Pax7 expression in human MuRC, performed transcriptomic analysis on MuRC subpopulations and characterized their metabolic and bioenergetic profile. We identified two quiescent MuRC states distinguished by relative Pax7 expression: Pax7^High^ MuRC, with stemness properties, and Pax7^Low^ MuRC, more committed to myogenic differentiation. We further show that Pax7^High^ MuRC are in a deeper quiescent state (dormancy) and operate a metabolic shift toward FAO. This study is the first report of transcriptomic analysis of human MuRC subpopulations based on Pax7 expression. Our findings strongly suggest that human Pax7^High^ MuRC are in a deeper quiescent state, less committed to differentiation and displaying a reduced metabolic activity. These data underline that human Pax7^High^ MuRC may constitute an appropriate stem cell source for potential therapeutic applications in skeletal muscle diseases.

## Methods

### Isolation of human primary myoblasts

Human muscle biopsies were collected during orthopedic surgery of healthy patients without known muscular diseases and processed as previously described [33]. Briefly, muscles were enzymatically dissociated using trypsin–EDTA 0.05% (Thermo Fisher, St. Louis, MO, USA) and freshly dissociated mononucleated cells were expanded for 5 to 7 days in a growth medium (GM) consisting of Ham’s F10 (Thermo Fisher) supplemented with 15% FCS (Thermo Fisher), bovine serum albumin (Sigma-Aldrich, St. Louis, MO, USA; 0.5 mg/ml), fetuin (Sigma-Aldrich; 0.5 mg/ml), epidermal growth factor (Life Technologies; 10 ng/ml), dexamethasone (Sigma-Aldrich; 0.39 μg/ml), insulin (Sigma-Aldrich; 0.04 mg/ml), creatine (Sigma-Aldrich; 1 mM), pyruvate (Sigma-Aldrich; 100 μg/ml), uridine (Sigma-Aldrich; 50 μg/ml) and gentamycin (Thermo Fisher; 5 μg/ml).

Human myoblasts (CD56 + /CD146 + /CD82 +) were sorted by flow cytometry using a FACSAria2 (BD Biosciences, New Jersey, USA), amplified in GM and used for experiments between 2 and 6 passages (less than 30 cell divisions).

### In vitro generation of human MuRC

Human myoblasts, at 80% of confluency, were switched to a differentiation medium (DM, composition detailed in [31]) for 24 h to 96 h. Human muscle reserve cells (MuRC) were then separated from myotubes after short trypsinization and filtration using 20 μm pre-separation filters (Miltenyi Biotec, Bergisch Gladbach, Germany), after 48 h in DM (48 h-MuRC), after 72 h in DM (72 h-MuRC) or after 96 h in DM (96 h-MuRC).

### Cell number and cell size

Freshly isolated human myoblasts, 48 h-MuRC and 96 h-MuRC were washed twice in PBS and resuspended in PBS. Cell number and cell size were determined using the automatic cell counter CellDrop BF (DeNovix, Wilmington, USA).

### qRT-PCR

RNA extraction (myoblasts, 48 h-MuRC and 96 h-MuRC) was performed with TRIzol reagent (Life Technologies) according to the manufacturer's protocol. After measuring sample concentrations, 0.5 µg of total RNA was reverse-transcribed with the PrimeScript™ RT reagent Kit (TaKaRa) according to the manufacturer's instructions. qRT-PCR experiments were all performed at the iGE3 Genomics Platform of the University of Geneva (http://www.ige3.unige.ch/genomics-platform.php) on an SDS 7900 HT instrument (Applied Biosystems). Raw threshold-cycle (Ct) values obtained with SDS 2.2 (Applied Biosystems) were imported into Excel. Normalization factor were calculated using the geNorm method [34]. Primers used for qRT-PCR were: Hs β2-microglobulin forward, 5′-TGCTCGCGCTACTCTCTCTTT-3′; Hs β2-microglobulin reverse, 5′-TCTGCTGGATGACGTGAGTAAAC-3′; Hs EEF1A1 forward, 5′-AGCAAAAATGACCCACCAATG-3′; Hs EEF1A1 reverse, 5′-GGCCTGGATGGTTCAGGTA-3′; Hs Pax7 forward, 5′- AAACACAGCATCGACGGCA-3′; Hs Pax7 reverse, 5′- CTCGTCCAGCCGGTTCC-3′; Hs MyoD forward, 5′- TGCCACAACGGACGACTTC -3′; Hs MyoD reverse, 5′- CGGGTCCAGGTCTTCCGAA-3′; Hs HES1 forward, 5′- CAGATGACGGCTGCGCT -3′; Hs HES1 reverse, 5′- TCGGTACTTCCCCAGCACAC-3′.

### Western blot

Total protein extract was obtained by harvesting human myogenic cells after expansion in GM (MB) or after differentiation for 24 h in DM. After 48 h to 96 h in DM, myotubes can be separated from MuRC after short trypsinization and 20 μm filtration to obtain myotubes fractions or MuRC fractions after 48 h, 72 h or 96 h in DM. Cells were centrifugated and cell pellets were rinsed twice with PBS containing phosphatases inhibitors (PhosSTOP, Sigma-Aldrich, cat#04906837001) and immediately lysed in CHAPS 1%-PhosSTOP 1X containing protease inhibitors (Complete tablet, Sigma-Aldrich, cat#04693124001) on ice for 5 min. Cell lysates were centrifugated for 5 min at 13′000 rpm, and supernatant protein content was dosed using a Bradford assay. Total proteins were separated on a 10% SDS–polyacrylamide gel (8% for detection of ACC and phosphor-ACC) and transferred to nitrocellulose membranes (Macherey–Nagel, Düren, Germany). Membranes were saturated in Tween/Tris-buffered saline (0.1% Tween 20, 20 mmol/l Tris–HCl (pH 7.5), and 137 mmol/l NaCl)-PVA 1X (Sigma-Aldrich) and incubated with the primary antibodies diluted in TTBS-3% BSA overnight at 4 °C as follows: rabbit anti-NICD (1/300, Cell Signaling), mouse anti-human Pax7 (1:300, DSHB), rabbit anti-MyoD (1:500, Cell Signaling), rabbit anti-AMPK⍺1 (1:2000, Abcam, cat#32,047), rabbit anti-phospho-ACC (1:1000, Cell Signaling cat# 11818S), rabbit anti-ACC (1:1000, Cell Signaling, cat#3662S, 1:1000) and mouse anti-α-tubulin (1:6000, Sigma-Aldrich). Membranes were then washed 3 times with TTBS for 10 min and incubated for 1 h with HRP-conjugated goat anti-mouse (1:10,000, BioRad, Hercules, CA, USA) and/or HRP-conjugated goat anti-rabbit antibodies (Cell Signaling, cat#7074S, 1/10000). Blots were revealed using ECL reagents (PerkinElmer) and mxECL Imager (Thermo Scientific). Image analysis was performed using FiJi software (ImageJ) to quantify the level of protein expression.

### Glycolytic capacity and mitochondrial function of human myoblasts and human MuRC

Oxygen consumption rate (OCR, pm/min) for the mitochondrial function and extracellular acidification rate (ECAR, mpH/min) for the glycolytic capacity were measured using a Seahorse XFe96 Analyzer (Agilent Technologies, Santa Clara, CA, USA) and performed at the READS unit, University of Geneva. Human myoblasts, 48 h-MuRC and 96 h-MuRC were freshly isolated as previously described and seeded in an XF96 V3 PS Cell Culture Microplate (Agilent Technologies, cat# 101,085–004) in GM for myoblasts (12,000 cells /well) and in DM for MuRC (20,000 cells /well) 24 h before the assay. Prior to the calibration step, the sensor cartridge was loaded with drugs (Agilent Technologies) and incubated for 1 h at 37 °C with XF Calibrant (Agilent Technologies, cat# 100,840-100) in a CO_2_-free incubator. Before the acquisition, cells were washed twice with a modified KRBH buffer (140 mM sodium chloride; 3.6 mM potassium chloride; 0.5 mM sodium phosphate monobasic; 0.5 mM magnesium sulfate; 1.5 mM calcium chloride and 10 mM HEPES; pH = 7.4).

The bioenergetic profile of the cells was evaluated using the Seahorse XF Glycolysis Stress Test Kit (Agilent Technologies, cat# 103,020-100) by sequential additions of 10 mM glucose, 1 μM oligomycin and 50 mM 2-deoxy-glucose (2-DG), or with the Seahorse XF Cell Mito Stress Test Kit (Agilent Technologies, cat# 103,015-100) by sequential additions of 1 μM oligomycin, 2 μM of carbonyl cyanide 4-(trifluoromethoxy) phenylhydrazone (FCCP) and 0.5 μM of rotenone/antimycin A.

Extracellular acidification rate (ECAR, glycolysis stress test) and oxygen consumption rate (OCR, mitochondrial stress test) were measured every 5 min over 120 min. Hoechst 33,342 (Invitrogen, cat#C10640G, 20 µg/ml) was added at the end of the experiment with the last drug injection, to normalize the results to the total cell number per well. Pictures were acquired using a Cytation 5 imaging reader (Agilent Technologies), and analysis was performed using Gen5 software (Agilent Technologies). Data normalization was performed on Wave software (Agilent Technologies) and analyzed with Microsoft Excel.

### Flow cytometry

#### Pax7 intra-cellular staining

Freshly isolated human myoblasts, 48 h-MuRC and 96 h-MuRC were washed twice in PBS and stained with Fixable Viability Stain (FVS) 780 (1:1000, BD Biosciences, cat#565,388) for 10 min at room temperature (RT) to exclude dead and apoptotic cells. Cells were then washed 2 times with PBS and fixed/permeabilized using the Transcription Factor Buffer Set (BD Biosciences, cat#562,574). Human cells were incubated for 5 min at RT with Human TruStain FcX™ (BioLegend, cat#422,302, 5 µl/millions of cells) and stained with the following antibodies for 40 min at 4 °C: mouse anti-human CD56-BUV395 (BD Biosciences cat#740,299) and mouse anti-human Pax3/7 Alexa Fluor® 647 (Santa Cruz Biotechnology, Dallas, TX, USA, cat#sc-365843). Negative control samples received equivalent amounts of BUV395- and AlexaFluor647-labeled isotype-matched antibodies. Cells were then washed 3 times with Perm/Wash buffer and resuspended in 400 μl of PBS until flow cytometry acquisition.

#### RNA-level detection by Hoechst and pyronin Y

Freshly isolated human myoblasts or human 48 h-MuRC were resuspended in PBS at a concentration of 1 × 10^6^ cell/ml and incubated first with Hoechst 33,342 at 5 μg/ml (Thermo Fisher) for 45 min at 37 °C to avoid non-specific binding of pyronin Y to DNA. Cells were then incubated with pyronin Y (Sigma-Aldrich) at 1 μg/ml for 45 min at 37 °C and then kept at 4 °C until flow cytometry acquisition using a BD LSRFortessa.

#### 2-NBDG uptake and palmitate uptake

An adherent monolayer of human myoblasts in GM, in DM48h or in DM96h were incubated for 1 h at 37 °C in DMEM glucose-free and sodium pyruvate-free (Gibco, cat#11,966-025) containing 2-NBDG (Tocris Biosciences, Bristol, UK, cat#6065/5, 100 µM) or palmitic acid-Lissamine rhodamine (Avanti Polar Lipids, Birmingham, AL, USA, cat#810104P, 1 µM). Cells were washed 3 times with PBS, trypsinized and then incubated for 10 min at RT with Fixable Viability Stain (FVS, 1:1000) diluted in PBS. Cells were then incubated for 40 min at 4 °C with anti-CD56-BUV395 (BD Biosciences cat#740,299) and washed 3 times with PBS. An isotype BUV395 was used at the same concentration for the control.

All acquisitions were performed using a BD LSRFortessa analyzer and DIVA software at the flow cytometry core facility at, the University of Geneva. Analysis was performed using FlowJo 10.7.2 (FlowJo LLC, USA). For the median fluorescence intensity ratio (MFI ratio), we divided the median fluorescence intensity obtained with 2-NBDG or palmitate by the median fluorescence intensity obtained with the negative control.

### Bulk transcriptomics

#### RNA sequencing

Human myoblasts and human 48h-MuRC were generated from six different biological replicates (*N* = 6) and stained for Pax7 as previously described (Pax7 intra-cellular staining) in the presence of RNAse inhibitor (l/500, RNasin® Ribonuclease Inhibitors, Promega) in all buffers from the fixation step to the cell sorting step. Human myoblasts, MuRC-Pax7^High^ and MuRC-Pax7^Low^ subpopulations were then prepared for bulk RNA sequencing according to the following steps: (1) flow cytometry cell sorting of human myoblasts Pax7^+^, of MuRC-Pax7^High^ subpopulation and of MuRC-Pax7^Low^ subpopulation (200,000 cells per condition) using a BD FACS Aria2 collected in FACS tube containing RNAse inhibitor and RNA later (2) RNA was extracted using either a high pure FFPE RNA Isolation Kit (Roche, cat#06650775001) or an RNeasy FFPE kit (Qiagen). Both kits were used following the manufacturer’s protocol and kept at -80 °C until use (3) RNA concentrations were obtained using Qubit RNA assay (Life Technologies, New York, USA). The quality of the RNA (RNA integrity number) was determined on the Agilent 2100 Bioanalyzer (Agilent Technologies, Palo Alto, CA, USA). (4) Sequencing was performed on the HiSeq 4000 unit (Illumina, San Diego, CA) using Illumina TruSeq RNA Exome (previously known as the Illumina TruSeq RNA Access Library Prep). Samples were sequenced as single-end 75 bp, and an average of 21,769,230 reads were generated for each sample. RNA-seq experiments were performed at the iGE3 Genomics Platform of the University of Geneva (http://www.ige3.unige.ch/genomics-platform. php).

#### Analysis

The iGE3 Genomics Platform performed two independent analyses on each batch of samples. Reads were subjected to quality control using FastQC (Picard) and mapped using STAR program version 2.7.1b on the human gene (hg38). Sequences alignment and genes count were summarized using the Python software (HTSeq, v.0.9.1). Low-expressing genes were filtered independently, and we then assemble the different sets of data from the analyses of the different samples.

Unsupervised analyses by principal component analysis (PCA) and supervised analyses were conducted in R program version 4.0.2 on log2 transformed gene expression. Differential expression analysis was then performed with the R package edgeR. Data were processed with R language, and graphs were produced with R language.

Data were analyzed using R program version 4.0.2 and are expressed as log2 fold change (Log2FC), adjusted p-value. The lists of genes differentially expressed between Pax7^High^ and Pax7^Low^ or Pax7^High^, and myoblasts were obtained as follows. A cut-off at *P* < 0.01 was applied with a Log2FC of 0.5. In addition, the Log2FC was required to be above 0.5 for a least four donors.

### Immunofluorescence staining

Adherent cultures of human myoblasts in GM or in DM for 48 h, on coverslips, were fixed for 15 min with PBS-4% PFA and washed 3 times in PBS. For primary cilia staining, cells were fixed 15 min with PBS-1% PFA, washed 3 times in PBS and then incubated for 2 min with -20 °C methanol. Cells were then permeabilized and blocked for 30 min at RT using the following blocking solution: PBS—2% BSA—5% goat serum (GS)—0.3% Triton. Cells were then incubated with the following primary antibodies diluted in the blocking solution, overnight at 4 °C: rabbit anti-acetyl-α-Tubulin (Cell Signaling, cat#5335, 1:1000), mouse anti-Pax7 (1:100, DSHB, Iowa, USA) and rabbit anti-MyoD (1:500; Cell Signaling). Cells were washed 3 times in PBS and incubated for 5 min at RT in the blocking solution. Cells were finally incubated with the following secondary antibodies diluted in blocking solution for 90 min at RT: goat anti-mouse IgG AlexaFluor488 (Life Technologies, cat#A11029, 1:1000) and goat anti-rabbit IgG AlexaFluor546 (Life Technologies, cat#A11035, 1:1000)), and the nuclei were stained with DAPI (0,3 µM). After incubation, cells were washed 3 times with PBS, and coverslips were mounted on glass slides using Immu-Mount™ (Expredia, #cat 9,990,402). Images were acquired using a Zeiss Axio Imager Z1 microscope. Nine random fields were acquired in each condition. Image analysis was performed using FiJi software (ImageJ).

### Statistical analysis

GraphPad Prism version 9.5.1 for macOS was used for all the statistical analyses. Results are expressed as mean ± SEM with n the number of biological replicates. Experiments were performed with a minimum of three biological replicates as indicated in the figure legend.

A one- and two-way analysis of variance (ANOVA) test followed by Tukey’s multiple comparisons test was used to compare more than two groups of samples. Statistical significance between two groups was calculated by a two-tailed Student’s t test. When the Gaussian distribution of the data could not be established using normality tests, Mann–Whitney nonparametric rank test was used. A 0.05 level of confidence was considered significant, *P < 0.05, **P < 0.01, ***P < 0.001, ****P < 0.0001.

## Results

### Notch signaling is activated and required for the generation of human MuRC in vitro

Human primary myoblasts were expanded in growth medium (GM) until 80% of confluency and switched to differentiation medium (DM) for 24 h, 48 h, 72 h or 96 h. Myoblasts in GM (MB), myotubes + MuRC after 24 h in DM (DM 24 h), MuRC (48 h, 72 h or 96 h) and myotubes (48 h, 72 h or 96 h) were then isolated and prepared for qRT-PCR (only MB, 48 h-MuRC and 96 h-MuRC) and Western blot analysis.

**Fig. 1:**
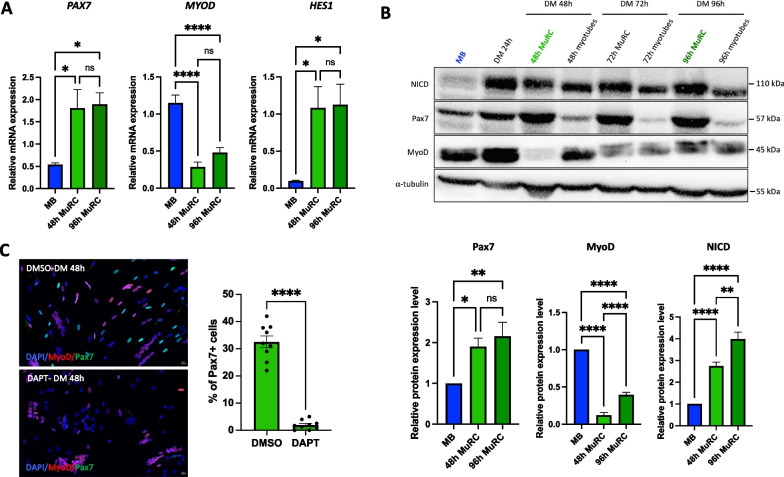
Notch signaling is activated and required for the generation of human MuRC in vitro. **A** Total mRNA were isolated from human myoblasts in growth medium (MB), 48 h MuRC and 96 h MuRC. qRT-PCR of Pax7, MyoD and the Notch target gene, Hes1. Results are presented as mean ± SEM.; *n* = 6, one-way ANOVA. **B** Total protein extracts were isolated from human myoblasts in growth medium (MB) or after 24 h in DM. MuRC and myotubes fractions were separated after 48 h, 72 h or 96 h in DM. Western blot analysis for Pax7, MyoD and the notch intra-cellular domain (NICD). Results are presented as mean ± SEM for myoblasts (MB), 48 h MuRC and 96 h MuRC; *n* = 6, one-way ANOVA. Full-length blots are presented in Additional file 1: Fig. S1 **C** Immunofluorescence staining for Pax7 (green), MyoD (red) and DAPI (blue) of human myogenic culture after 48 h in differentiation medium (DM) in the presence of DMSO or the Notch inhibitor, DAPT. The images shown are representative of the three independent experiments. Results are presented as mean ± SEM; *n* = 3 with 3 images analyzed per experiment, unpaired t test. Scale bar: 10 μm

*PAX7* and *HES1* mRNA levels were significantly higher in human MuRC as compared to proliferating human myoblasts with no significant differences between 48 h-MuRC and 96 h-MuRC (Fig. 1A). In contrast, expressions of *MYOD* mRNA levels were significantly decreased in MuRC as compared to myoblasts with no significant difference between 48 h- and 96 h-MuRC.

At a protein level, we revealed that Pax7 and NICD (notch intra-cellular domain) expressions were higher in human MuRC as compared to myoblasts (Fig. 1B). Moreover, we noticed that NICD expression was significantly higher in 96-MuRC as compared to 48 h-MuRC. Inversely, MyoD expression was significantly reduced in human MuRC as compared to myoblasts. We also observed that 96 h-MuRC expressed higher levels of MyoD as compared to 48 h-MuRC. Finally, in the presence of DAPT (a notch inhibitor) during myogenic differentiation (DAPT-DM48h), we drastically decreased the in vitro generation of Pax7^+^ human MuRC with only 2.0 ± 0.6% of Pax7^+^ MuRC as compared to 32.6 ± 2.5% of Pax7^+^ MuRC in DMSO-DM48h (mean ± SEM, *n* = 3, Fig. 1C). Together, these results reveal that Notch signaling is active and essential for the generation of human Pax7^+^ MuRC in vitro.

### Human MuRC are transcriptionally less active and possess a primary cilium

**Fig. 2:**
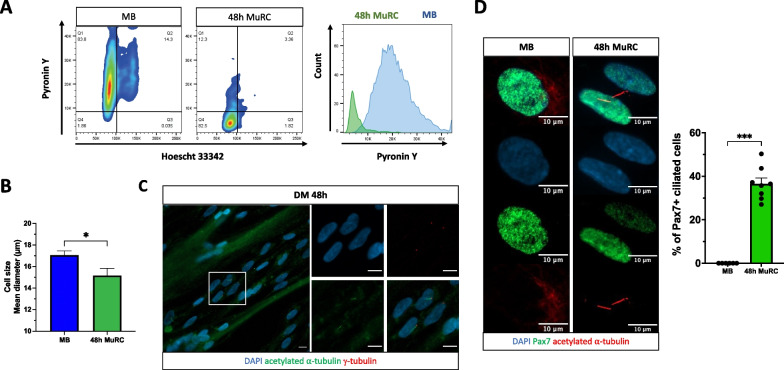
Human MuRC are transcriptionally less active and possess a primary cilium. **A** Human myoblasts and 48 h-MuRC were stained with Hoescht 33,342 and pyronin Y and analyzed by flow cytometry. One histogram is shown, representative of three independent experiments. **B** Cell size of human myoblasts and of 48 h-MuRC, measured immediately after trypsinization, mean ± SEM, *n* = 6, one-way ANOVA. **C** Immuno-detection of primary cilia on myogenic culture after 48 h in DM and stained with antibodies against acetylated α-tubulin and γ-tubulin. **D** Immuno-detection of primary cilia on human myogenic cells in different cellular states; proliferating myoblasts (MB) and quiescent MuRC (48 h-MuRC). Staining with antibodies against Pax7 and acetylated α-tubulin (mark cilia) and DAPI. Values shown on the histogram are mean ± SEM; *n* = 8, unpaired t test

To further characterize the quiescent state of human MuRC (48 h-MuRC), we monitored transcript dynamics in living cells using pyronin Y, an RNA intercalator. We observed that MuRC exhibit significantly reduced pyronin Y levels as compared to myoblasts (Fig. 2A), confirming the quiescent status of MuRC. This drop in RNA content goes together with a significant decrease in MuRC cell size as compared to myoblasts (Fig. 2B). We also examined if human MuRC possess a primary cilium, a structure that was described in quiescent MuSC, preferentially in cells committed to self-renewal [18, 35]. Using specific antibodies directed against γ-tubulin and acetylated α-tubulin, we observed that a proportion of MuRC, located between myotubes after 48 h in differentiation medium (DM48h), possess a primary cilium (Fig. 2C). We then stained myoblasts or MuRC using Pax7 and acetylated α-tubulin antibodies, and we showed that 38% of Pax7^+^ human MuRC possess a primary cilium, whereas we could not detect any cilia in Pax7^+^ proliferating myoblasts (Fig. 2D). Together, these results reveal that human MuRC are a heterogeneous population with a small proportion of cells (38%) that express a primary cilium.

### Human MuRC are heterogeneous for Pax7 expression

**Fig. 3:**
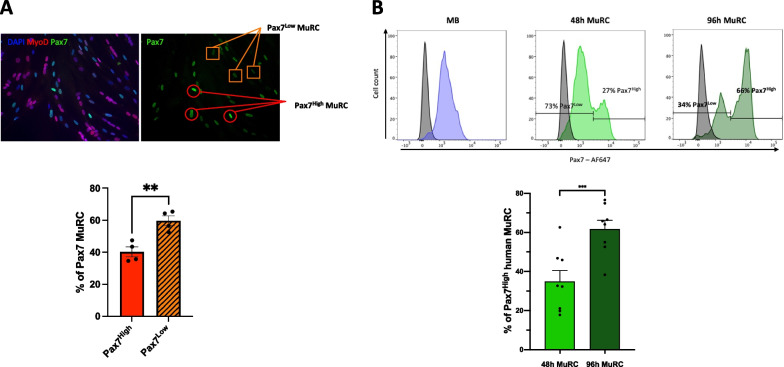
Human MuRC are heterogeneous for Pax7 expression. **A** Immunofluorescence staining for Pax7 (green), MyoD (red) and DAPI (blue) of human myogenic cells after 48 h in differentiation conditions (48 h DM). Orange squares identify Pax7^High^ MuRC and red circles identify Pax7^Low^ MuRC. Quantification of Pax7^High^ MuRC and Pax7^Low^ MuRC after immunofluorescence staining is presented as mean ± SEM; *n* = 4, unpaired t test. **B** Flow cytometry analysis of human myoblasts (blue) and of human MuRC isolated after 48 h (light green) or 96 h (dark green) in DM, fixed, permeabilized and stained with antibody against Pax7 and CD56. The isotype control staining for Pax7 is presented in black for all analysis. The histogram represents the percentage of Pax7^High^ human MuRC after 48 h or 96 h in DM. Results are presented as mean ± SEM; *n* = 8, unpaired t test

By using antibodies directed against Pax7 and MyoD, we observed by immunofluorescence that human MuRC are Pax7^+^/MyoD^−^ myogenic cells. Interestingly, we noticed that Pax7 expression was heterogeneous in the pool of human MuRC. By immunofluorescence, we revealed a Pax7^High^ subpopulation that represents 40.3 ± 3.0% of the MuRC population (orange square, Fig. 3A) and a Pax7^Low^ subpopulation that represents 59.7 ± 3.0% of the MuRC population (red round, mean ± SEM, *n* = 4, Fig. 3A). By flow cytometry, we further confirmed these results. After Pax7 staining (Fig. 3B), we identified two distinct MuRC subpopulations based on Pax7 expression, a Pax7^High^ and a Pax7^Low^ subpopulation. In 48 h-MuRC, the Pax7^High^ subpopulation represents 34.9 ± 5.6% of the total MuRC population, whereas in 96 h-MuRC, the Pax7^High^ subpopulation represents 61.7 ± 4.4% of the total MuRC population (mean ± SEM, *n* = 8, Fig. 3B). Of note, there is also a strong heterogeneity in the value of the ratio Pax7^High^/Pax7^Low^ between the eight biological replicates (Fig. 3B).

### Bulk RNA-seq analysis of human MuRC subpopulations based on PAX7 expression

**Fig. 4:**
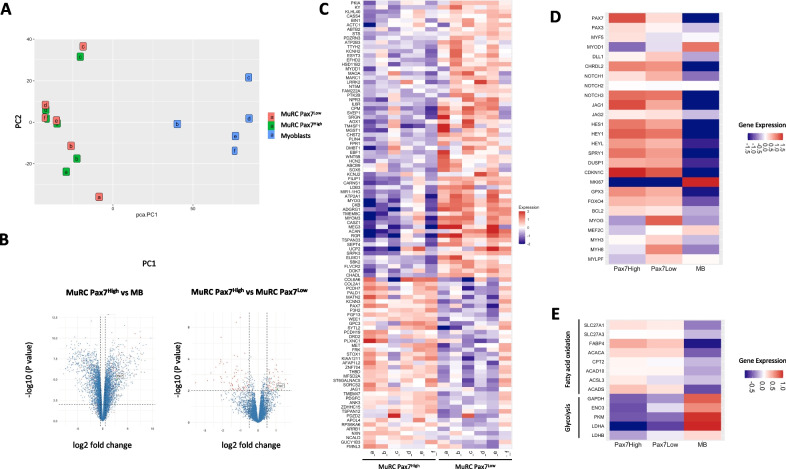
Transcriptional profiles of human Pax7^High^ and Pax7^Low^ MuRC subpopulations. All human cells (myoblasts, MuRC-Pax7^High^ and MuRC-Pax7^Low^) were fixed/permeabilized and stained for Pax7 before flow cytometry and mRNA isolation. **A** Biological samples were represented by principal component analysis (PCA). PC1/PC2 analysis demonstrated that human MuRC and human myoblasts displayed very different expression profiles (PC1), whereas for each biological sample analyzed by RNA-seq, we observed that Pax7^High^ and Pax7^Low^ MuRC are very similar in gene expression profile. **B** Volcano plot of the supervised analysis comparing myoblasts and Pax7^High^ MuRC and comparing Pax7^High^ MuRC and Pax7^Low^ MuRC transcriptomes. Strong transcript modifications between myoblast and Pax7^High^ MuRC were observed with 3393 modified genes whereas the changes were moderate between Pax7^High^ MuRC and Pax7^Low^ MuRC with 103 modified genes (Log2FC > 0.5, *P* < 0.01). **C** Heatmap showing the number of normalized reads of MuRC-RNA-seq data for the 103 modified genes between Pax7^High^ MuRC and Pax7^Low^ MuRC populations. (**D**, **E**) Heatmap showing the average number of normalized reads of myoblasts (*n* = 5), of Pax7^High^ MuRC (*n* = 6) and of Pax7^Low^ MuRC (*n* = 6) RNA-seq data for a selection of genes

We compared the transcriptional profile of human myoblasts, Pax7^High^ MuRC and Pax7^Low^ MuRC using bulk RNA-seq (6 biological replicates). Of note, all cells (myoblasts and 48 h-MuRC) were fixed/permeabilized and stained for Pax7 before flow cytometry isolation to ensure comparability between all RNA preparations. mRNA preparation from fixed/permeabilized cells did not interfere with RNA integrity and quality. The quality control check done on raw sequence data using FastQC was good and the alignment percentage of mapped reads was correct (84% on average). The normalization and differential expression analysis were performed with the R package edgeR, and samples were represented by principal component analysis (PCA). PC1/PC2 analysis demonstrated that human MuRC and human myoblasts displayed very different expression profiles (PC1), whereas for each biological sample analyzed, we observed that Pax7^High^ and Pax7^Low^ MuRC are very similar in gene expression (Fig. 4A). A volcano plot of the supervised analysis comparing myoblasts, Pax7^High^ MuRC and Pax7^Low^ MuRC transcriptomes demonstrated that 3393 genes showed strong transcript modifications between myoblasts and Pax7^High^ MuRC (Fig. 4B). We also found that only 103 genes showed strong transcript modifications between Pax7^High^ MuRC and Pax7^Low^ MuRC. These genes are involved in various pathways and include the transcription factor Pax7, which validates our RNA-seq approach (Fig. 4C).

The comparison between Pax7^High^ MuRC, Pax7^Low^ MuRC and myoblasts transcriptomes highlighted various genes related to quiescence that are upregulated in human MuRC as compared to human myoblasts (Fig. 4D). In agreement, expression of the proliferation marker *KI67* is downregulated in human MuRC as compared to human myoblasts. Moreover, *JAG1* and the downstream targets of Notch signaling *HES1* and *HEYL* were increased in Pax7^High^ MuRC as compared to Pax7^Low^ MuRC. Similarly, the quiescent-associated genes *SPROUTY1* and the cyclin-dependent kinase inhibitor 1C (*CDKN1c*) were found upregulated in Pax7^High^ MuRC, supporting a deeper quiescent state of Pax7^High^ MuRC as compared to Pax7^Low^ MuRC. In agreement, expression of the muscle regulatory factor *MyoD1* and the myogenic differentiation markers myogenin, *MEF2C* and myosin heavy chain (*MYH3*, *MYH8* and *MYLPF*) was downregulated in Pax7^High^ MuRC as compared to Pax7^Low^ MuRC. We also noticed that various genes implicated in the resistance to oxidative stress (*GPX3* or *FOXO4*) and implicated in apoptosis (*BCL2)* were upregulated in MuRC (Fig. 4D). The RNA-seq data also demonstrated differential expression of genes regulating metabolism between human MuRC and myoblasts. We noticed that gene expression related to fatty acid oxidation (FAO) (*SLC27A1, SLC27A3, ACACA, ACADS…*) were upregulated in human MuRC, whereas genes involved in glycolysis (*GAPDH, ENO3, PKM, LDHA/B*) displayed lower expression in MuRC as compared to myoblasts (Fig. 4E).

### Human MuRC significantly reduced glycolysis, maximal respiration and ATP-linked respiration as compared to myoblasts

**Fig. 5:**
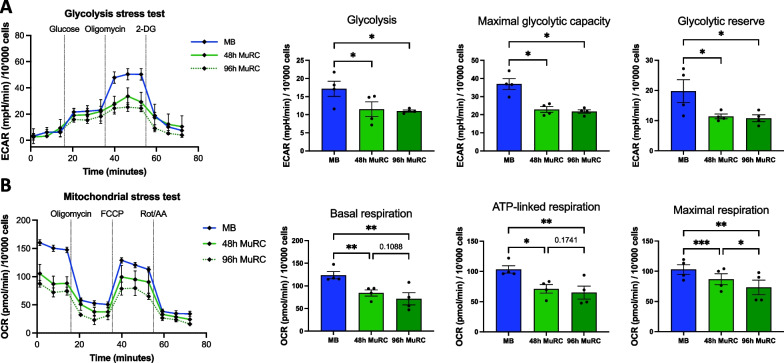
Human MuRC significantly reduced glycolysis, maximal respiration and ATP-linked respiration as compared to myoblasts. ECAR (extracellular acidification rate) and OCR (oxygen consumption rate) were assessed in human myoblasts and in human MuRC isolated after 48 h or 96 h in DM (48 h-MuRC or 96 h-MuRC). **A** After the evaluation of glycolysis post-glucose injection, oligomycin and 2-deoxy-glucose (DG) were added as shown (*n* = 4). Glycolysis, maximal glycolytic activity and glycolytic reserve were measured for human myoblasts, 48 h-MuRC and 96 h-MuRC (mean ± SEM, *n* = 4, one-way ANOVA). **B** After the evaluation of basal respiration, oligomycin, FCCP (carbonyl cyanide 4-(trifluoromethoxy) phenylhydrazone) and ROT/AA (rotenone/antimycin A) were added as shown (*n* = 4). Basal respiration, ATP-linked respiration and maximal respiration were measured for human myoblasts, 48 h-MuRC and 96 h-MuRC (mean ± SEM, *n* = 4, one-way ANOVA). Seahorse data were normalized to the number of cells per well

Pax7^High^ MuRC are enriched in the 96 h-MuRC population as compared to the 48 h-MuRC population with respectively 62% and 35% of Pax7^High^ cells. Therefore, we investigated the glycolytic activity and mitochondrial respiration of 48 h-MuRC and 96 h-MuRC as compared to myoblasts. We estimated the cellular glycolytic activity by assessing the extracellular acidification rate (ECAR). A significant decrease in ECAR was observed for MuRC as compared to myoblasts with a significant reduction in glycolysis, maximal glycolytic capacity and glycolytic reserve in MuRC as compared to myoblasts. We observed no significant ECAR difference between 48 h-MuRC and 96 h-MuRC (Fig. 5A). We then measured the oxygen consumption rate (OCR), an indicator of mitochondrial oxidative activity. We observed that human MuRC reduced their basal respiration, ATP-linked respiration and maximal respiration as compared to human myoblasts (Fig. 5B). Of note, 96 h-MuRC have a significantly reduced maximal respiration and a tendency to lower basal respiration and ATP-linked respiration as compared to human 48 h-MuRC.

### Human MuRC overexpressed active AMPKα1 in correlation with increased fatty acid uptake

AMP-activated kinase (AMPK) is an energy sensor that regulates cellular metabolism and phosphorylates diverse target enzymes including the acetyl-CoA carboxylase (ACC). We thus quantified AMPKα1 expression in human MuRC (48 h and 96 h) and evaluated its activity through quantification of the phospho-ACC, known to promote the entry of fatty acid into mitochondria.

**Fig. 6:**
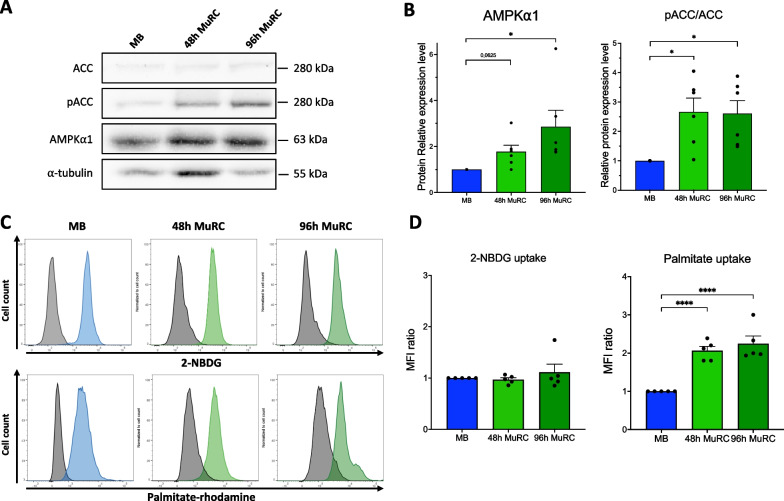
Human MuRC overexpressed active AMPKα1 in correlation with increased fatty acid uptake. **A** Western blot analysis of AMPKα1, acetyl-CoA carboxylase (ACC) and phospho-ACC (pACC) in human myoblasts (MB), human 48 h-MuRC and 96 h-MuRC. Full-length blots are presented in Additional file 1: Fig. S2. Quantifications are given in (**B**) after the normalization of protein level to α-tubulin. Data are presented as mean ± SEM; *n* = 6, one-way ANOVA. **C** Representative histogram after flow cytometry analysis of glucose (2-NBDG) and fatty acid (palmitate rhodamine) uptake by human myoblasts (MB), 48 h-MuRC and 96 h-MuRC. **D** Quantification of the median fluorescence intensity (MFI) ratio for 2-NBDG and palmitate uptake of five independent experiments. Data are presented as mean ± SEM; *n* = 5, one-way ANOVA

By western blot, we observed an increase in AMPKα1 expression and of pACC/ACC ratios, in MuRC as compared to myoblasts. When normalized to α-tubulin and compared to myoblasts, we noticed that 48 h-MuRC and 96 h-MuRC increase AMPKα1 expression by 1.7-fold and 2.8-fold, respectively. pACC/ACC ratios were also higher in MuRC as compared to myoblasts with a 2.6-fold and a 2.7-fold increase, respectively (Figs. 6A and 6B). These data reveal that AMPKα1 is overexpressed and active in human MuRC. We further evaluated the capacity of human myoblasts and of human MuRC to uptake either glucose (2-NBDG) or fatty acid (palmitate rhodamine). Palmitate uptake strongly increase in 48 h-MuRC and 96 h-MuRC as compared to myoblasts with a twofold and 2.2-fold increase, respectively, whereas no change was noticed for 2-NBDG uptake (Figs. 6C and 6D). These data indicate that active AMPKα1 promote fatty acid uptake in human MuRC.

## Discussion

Emerging pieces of evidence have suggested a crucial role of the quiescent MuSC state associated with a higher level of Pax7 expression in the success of MuSC transplantation in mice [14, 29]. Nevertheless, freshly isolated MuSC are quickly activated out of quiescence during their isolation and amplification in vitro*,* which correlates with a drastic diminution of Pax7 expression and a significant reduction in their regenerative potential [29, 36]. We previously demonstrated that human MuRC, generated in vitro, are Pax7^+^ myogenic stem cells, able to survive and to participate in the muscle regeneration process after transplantation in mice [31]. In this study, we demonstrated for the first time that the pool of quiescent human MuRC is heterogeneous for Pax7 expression with a Pax7^High^ subpopulation that displays characteristics of dormancy and fulfills many criteria favorable for a possible translation to therapeutic application.

Human MuRC are known to escape terminal differentiation in vitro, to acquire a quiescent G_0_ state and preserve their ability to re-enter the cell cycle to give rise to a healthy progeny, the myoblasts. In this study, we observed that Notch signaling activity is increased in MuRC as compared to myoblasts and is also significantly higher in 96-MuRC as compared to 48 h-MuRC. In the presence of DAPT, a Notch inhibitor, the capacity of human myoblasts to escape terminal differentiation in vitro and to give rise to quiescent Pax7^+^ MuRC was nearly abolished. In agreement with other studies, our results confirm the important role of the Notch signaling pathway in actively promoting the generation of human MuRC through the regulation of Pax7 expression [37–39]. We also described, in human MuRC, a set of properties that are associated with quiescent MuSCs. We found that human MuRC displayed a slight, but significant, decrease in cell size relative to myoblasts. Consistent with this finding, human MuRC have a lower level of mRNA than myoblasts. We also observed that human MuRC are heterogeneous for the presence of a primary cilium (detected on 38% of human MuRC), a primary cilium that is lost upon MuRC activation (almost undetectable on human myoblasts) as described previously for murine MuSCs [18]. Moreover, the primary cilium is critical for maintaining MuSC regenerative capacity [21] and is implicated in the regulation of MuSC self-renewal [20]. Therefore, human MuRC expressing primary cilia may represent a subpopulation with higher self-renewal and regenerative capacities.

We also revealed that the quiescent human MuRC pool is molecularly and phenotypically heterogeneous. Based on the expression profile of Pax7, we demonstrated that the pool of human MuRC is composed of two distinct subpopulations: a Pax7^High^ MuRC and a Pax7^Low^ MuRC. These data are in accordance with previous studies that described Pax7 heterogeneity in murine MuSC [14]. Interestingly, Barruet et al. also identified and separated distinct human MuSC subpopulations with distinct functionality [15]. In our model, we also observed that the proportion of Pax7^High^ MuRC increased with time under differentiation conditions, rising from 35% after 48 h in DM to 62% after 96 h in DM (Fig. 3). We suggest that the increased ratio of Pax7^High^/Pax7^Low^ populations in 96 h-MuRC is rather related to the loss of part of the Pax7^Low^ population with time in DM rather than an increase in the Pax7^High^ population. Indeed, and consistent with the fact that the proportion of myotubes increases in human myogenic cultures with time in DM (from 55% after 48 h in DM to 70% after 96 h in DM [31]), we suggest that Pax7^Low^ 48 h-MuRC, more primed to myogenic differentiation (Fig. 4), are more likely to activate and fuse with existing myotubes than Pax7^High^ 48 h-MuRC during myogenic differentiation that occur between 48 and 96 h in DM.

We then performed bulk RNA sequencing on myoblasts, Pax7^High^ MuRC and Pax7^Low^ MuRC. We found that genes implicated in the Notch signaling pathway including *HES1*, *HEY1*, *HEYL* and *JAG1* positively correlate with Pax7 expression across the human MuRC pool. Moreover, the quiescent state of human MuRC seems to be associated with genes related to stress resistance including the enzyme *GPX3*, the gene *FOXO4* and the anti-apoptotic gene *BCL2* [40]. We also observed a higher expression of the cell cycle inhibitor gene *CDKN1c* in Pax7^High^ MuRC as compared to Pax7^Low^ MuRC and a lower expression of various myogenic differentiation markers. Together, these results strongly suggest that Pax7^High^ MuRC are in a deeper quiescent state (dormancy) and that Pax7^Low^ MuRC are in a quiescent state more primed to myogenic differentiation.

Quiescence is also known as a state of low cellular activity where limited demands for energy are associated with a low metabolic rate [23]. Our transcriptomic data showed that Pax7^High^ MuRC exhibit an enrichment of genes related to FAO and a decrease in genes involved in glycolysis, in agreement with the known metabolic switch from glycolysis to FAO taking place in quiescent MuSCs [41]. Accordingly, Seahorse experiments reveal that human MuRC decrease their glycolysis, glycolytic capacity, basal respiration and ATP-linked respiration as compared to myoblasts. Moreover, we showed a significant decrease in the maximal respiration of 96 h-MuRC as compared to 48 h-MuRC suggesting that Pax7^High^ MuRC, which represent 62% of the 96 h-MuRC population (Fig. 3), have reduced mitochondrial respiration as compared to Pax7^Low^ MuRC. We also showed an increased expression of active AMPK⍺1 in human MuRC that correlate with an increase fatty acid uptake. Together, these results demonstrate that human MuRC operate a metabolic shift from glycolysis toward FAO, a process known to contribute to the preservation of muscle stem cell quiescence [5, 22, 25, 42]. Metabolic factors are identified as playing a critical role in regulating MuSC fate decisions and functions that can also influence the efficacy of stem cell-based therapies [22, 41, 43, 44]. But whether and how cellular metabolism regulates the quiescent state of human MuSC or MuRC remain largely unknown. The substrate preference toward glucose, fatty acids or amino acids, originally considered as passive energy providers, has been shown to induce genetic reprogramming through the production of secondary metabolites in various stem cell types [45, 46]. Accordingly, metabolic flexibility of MuRC and its modulation can have major consequences on cell function and may be targeted to favor the generation of dormant MuRC subpopulations (Pax7^High^) with possible improved regenerative capacities.

## Conclusions

These data, which describe a set of properties that distinguish human MuRC from myoblasts, reveal that the quiescent MuRC pool is heterogeneous for Pax7 with a Pax7^High^ population in a deeper quiescent state, less committed to differentiation and with a lower metabolism. Altogether, our data suggest that human Pax7^High^ MuRC may constitute an appropriate stem cell source for potential therapeutic applications in skeletal muscle diseases.

### Supplementary Information

**Additional file 1. Figure S1. **Notch signaling is activated and required for the generation of human MuRC in vitro. Total protein extracts were isolated from human myoblasts in growth medium (MB) or after 24h in DM. MuRC and myotubes fractions were separated after 48h, 72h or 96h in DM. Western blot analysis for Pax7, MyoD and the notch intracellular domain (NICD). **Figure S2.** Human MuRC overexpressed active AMPKa1. Western blot analysis of AMPKa1, acetyl CoA carboxylase (ACC) and phosphoACC (pACC) in human myoblasts (MB), human 48h-MuRC and 96h-MuRC.

## Data Availability

The datasets used and/or analyzed during the current study are available from the corresponding author on reasonable request. The accession numbers for RNA-seq raw and processed files can be downloaded from the Gene Expression Omnibus website (https:// www.ncbi.nlm.nih.gov/geo/) under the accession number GEO: GSE233597.

## Abbreviations

ACC: Acetyl-CoA carboxylase
AMPK: AMP-activated protein kinase
DM: Differentiation medium
FAO: Fatty acid oxidation
GM: Growth medium
MuSC: Muscle stem cell
MuRC: Muscle reserve cells
