# Supplementary material for: In vitro-generated human muscle reserve cells are heterogeneous for Pax7 with distinct molecular states and metabolic profiles
Source: Stem Cell Res Ther. 2023 Sep 8;14:243. doi: 10.1186/s13287-023-03483-5 (PMC10486062; doi:10.1186/s13287-023-03483-5)
Supplement: Supplementary file 1 — Additional file 1. Figure S1. Notch signaling is activated and required for the generation of human MuRC in vitro. Total protein extracts were isolated from human myoblasts in growth medium (MB) or after 24h in DM. MuRC and myotubes fractions were separated after 48h, 72h or 96h in DM. Western blot analysis for Pax7, MyoD and the notch intracellular domain (NICD). Figure S2. Human MuRC overexpressed active AMPKa1. Western blot analysis of AMPKa1, acetyl CoA carboxylase (ACC) and phosphoACC (pACC) in human myoblasts (MB), human 48h-MuRC and 96h-MuRC. [file 13287_2023_3483_MOESM1_ESM.docx]

**Supplementary Figure 1: Full-length blots of Figure 1B.**

Notch signaling is activated and required for the generation of human MuRC in vitro. Total protein extracts were isolated from human myoblasts in growth medium (MB) or after 24h in DM. MuRC and myotubes fractions were separated after 48h, 72h or 96h in DM. Western blot analysis for Pax7, MyoD and the notch intracellular domain (NICD).

**Supplementary Figure 2: Full-length blots of Figure 6A.**

Human MuRC overexpressed active AMPKα1. Western blot analysis of AMPKα1, acetyl CoA carboxylase (ACC) and phosphoACC (pACC) in human myoblasts (MB), human 48h-MuRC and 96h-MuRC.
